# The Prevalence of Hypertension and Associated Risk Factors among Secondary School Teachers in Bahir Dar City Administration, Northwest Ethiopia

**DOI:** 10.1155/2021/5525802

**Published:** 2021-04-16

**Authors:** Destaw Damtie, Ayehu Bereket, Denekew Bitew, Bizuayehu Kerisew

**Affiliations:** ^1^Department of Biology, Bahir Dar University, Bahir Dar, Amhara Region, Ethiopia; ^2^Ghion Secondary School, Bahir Dar University, Bahir Dar, Amhara Region, Ethiopia; ^3^Department of Statistics, Bahir Dar University, Bahir Dar, Amhara Region, Ethiopia

## Abstract

**Background:**

Hypertension is one of the noncommunicable cardiovascular diseases (CVDs), and its prevalence is rising in middle- and low-income countries. It is not given enough attention in the developing countries like Ethiopia. Not enough data and studies about hypertension are available in Ethiopia. This study aimed to determine the prevalence of hypertension and its associated risk factors among secondary school teachers in Bahir Dar city administration.

**Methods:**

An institutional-based cross-sectional survey was conducted among secondary school teachers in Bahir Dar. Two hundred twenty-two randomly selected teachers were interviewed, and data related to the demographic, behavioral, health, and dietary characteristics of the individuals were recorded. Blood pressure data were taken. Logistic regression analysis had been used to assess independent risk factors for hypertension. *p*-values of less than 0.05 were considered statistically significant.

**Results:**

The overall prevalence of hypertension in the study was 29.28%. Age 41 to 50 (AOR: 2.506; 95% CI: 1.103–5.694; and *p*=0.028), having self-reported diabetes mellitus (AOR: 8.595; 95% CI: 2.795–26.424; and *p* < 0.0001), having a family history of hypertension (AOR: 3.387; 95% CI: 1.579–7.285; and *p*=0.002), khat chewing (AOR: 5.426; 95% CI: 1.811–16.256; and *p*=0.003), physical inactivity (AOR: 5.212; 95% CI: 1.974–13.763; and *p*=0.001), and presence of self-reported repeated stress (AOR: 3.027; 95% CI: 1.404–6.527; and *p*=0.005) were the risk factors associated with hypertension.

**Conclusions:**

Different intervention measures with a particular emphasis on prevention by introducing lifestyle modifications are highly recommended to mitigate and control hypertension.

## 1. Introduction

Blood pressure (BP) is the force of circulating blood on the walls of the arteries [1]. It has two components, namely, systolic BP (SBP) and diastolic BP (DBP) [2]. SBP is the maximum blood pressure during contraction of the ventricles, and DBP is the minimum pressure recorded before the next contraction [3]. BP is written with the SBP first, followed by the DBP (e.g., 120/80) [1]. Based on SBP/DBP, BP in adults is classified as normal (<120/<80), prehypertension (120–139/80–89), stage one hypertension (140–159/90–99), and stage two hypertension (≥160/≥100) [4]. Hypertension brings about the damage of the heart, damage of blood vessels in the brain and the kidneys, enlargement of the heart, heart failure, stroke, kidney failure, blindness, and cognitive impairment [5].

Globally, an estimated 17.9 million people died from cardiovascular diseases in 2016, representing 31% of all global deaths. Of these deaths, 85% are due to heart attack and stroke [6]. Hypertension is the leading risk factor for mortality and the main cause of global disability-adjusted life years (DALYs). In 2015, SBP attributed to 10.7 million deaths and nearly 212 million DALYs [7]. The prevalence of hypertension increase after 2000 may be due to lifestyle changes, and the highest global hypertension prevalence has shifted from high- and medium-income countries (HMIC) to low- and medium-income countries (LMIC) [8]. For example, in 2010, 28.5% of adult hypertension was in high-income countries and 31.5% in low- and middle-income countries [9]. Because of weak health systems, the number of people with hypertension who were undiagnosed, untreated, and uncontrolled is also higher in low- and middle-income countries compared to the high-income countries [10]. By 2025, the projected number of people with hypertension is expected to rise by 60% and reach 1.56 billion people globally [11].

Hypertension is a widespread problem in sub-Saharan Africa (SSA), and in some communities, it has been reported to be as high as 38% [12]. In 2015, the prevalence of hypertension among the Ethiopian population was estimated to be 19.6% (23.7% among urban population and 14.7% among the rural and urban combined population) (20.6% among males and 19.2% among females) [13].

Studies in different parts of Ethiopia also showed a high prevalence of hypertension in the country. The prevalence of hypertension in Jimma was 21.3% (22.2% in males and 19.6% in females) [14], 25.1% in Bahir Dar city [15], 16.45% [16] in Addis Ababa (19.13% among bankers and 21.8% among teachers) [17], and 27.9% in Dabat district and Gondar town (30.7% among urban and 25.3% among rural residents) [18].

There is no single precise cause of hypertension [19]. However, there are known risk factors that increase the possibility of hypertension. The risk factors of hypertension can be classified as modifiable and nonmodifiable [20]. Modifiable risk factors are risk factors of hypertension that many people can reduce their blood pressure by changing their diet and lifestyle [10]. They include unhealthy diets (excessive salt consumption, a diet high in saturated fat and trans fats, and low intake of fruits and vegetables), physical inactivity, consumption of tobacco and alcohol, and being overweight or obese, whereas the nonmodifiable risk factors of hypertension are a family history of hypertension, age over 65 years, gender, genetics, and coexisting diseases such as diabetes or kidney disease [21].

Many studies in Ethiopia have identified risk factors associated with hypertension, for example, the male gender, overweight, and sleep duration of ≤5 hours [22]; age, having ever smoked cigarette, the number of hours spent walking/cycling per day, the number of hours spent watching TV per day, history of diabetes, adding salt to food in addition to the normal amount that is added to the food during cooking, and body mass index [15]; having ever been told hypertensive, using animal product butter, physical inactivity, and BMI 25.0 to 29.9 and greater than 30 [23]; age, cigarette smoking, alcohol drinking, and khat chewing [14]; and tobacco use, alcohol abuse, overweight and physical inactivity together with poor access and utilization of health services, absence of health insurance schemes, and rapid urbanization [24].

Generally, different literatures showed that the prevalence of hypertension is increasing radically especially in developing countries like Ethiopia and particularly in urban settings. Its prevalence is associated with socioeconomic, demographic, lifestyle, and dietary factors. The present study thus aimed to evaluate the prevalence of hypertension and associated risk factors among secondary school teachers in Bahir Dar city administration.

## 2. Methods

### 2.1. Description of the Study Area

Bahir Dar is a town located in northwest Ethiopia at the southern extreme of Lake Tana where the Blue Nile starts its long journey to the Sudan and Egypt. It is situated at an altitude of about 1820 meters above sea level, at the geographic coordinates 11° 36′ N and 37° 25′ E [25]. The town is 567 and 465 km northwest away from Addis Ababa through Debre Markos–Bure and Dejen–Motta roads, respectively [26]. Based on the 2017/18 budget annual statistical bulletin of Amhara National Regional State Plan Commission, the city administration has a projected population of 341,608 (161,758 males and 179,850 females). From this population, 281,886 (82.5%) are urban inhabitants and the rest 59,720 (17.5%) rural inhabitants. The town has a total area of 361.74 square kilometers or 36,174.36 hectares. According to the information gathered from the city mayor office, the city administration is currently divided into six subcities, three satellite towns, and 26 urban and 14 rural “Kebeles” for administrative purposes (Kebele is the smallest administrative unit in Ethiopia). The three satellite towns/suburban areas are Zegie, Meshenti, and Tis Abay. The satellite towns have their municipalities, which are under the jurisdiction of the main city mayor. In the city administration, there are ten privately owned, ten government-owned, and one prison-owned secondary schools.

### 2.2. Study Design and Period

The researcher used an institutional-based cross-sectional descriptive survey to assess the prevalence and associated risk factors of hypertension among secondary school teachers in Bahir Dar city administration, northwest Ethiopia. All government-owned secondary schools were included. The secondary schools selected for the study had a total number of 710 teachers (492 males and 218 females). Interviews and physical measurements were conducted from December 2019 to February 2020. The gathered data were tabulated and statistically analyzed using SPSS software version 21.

### 2.3. Study Participants and Sampling Techniques

Ten government secondary schools of Bahir Dar city administration were included in the study. The complete name lists of teachers were collected from these schools. The collected lists of all teachers from these ten secondary schools were alphabetically ordered. Participants were selected by a computer-based systematic random sampling method. The required sample size was determined by Cochran's formula [27]:

(1) $$\begin{array}{c} n=z^{2}p\left(\frac{1-p}{e^{2}}\right), \end{array}$$

where *n* is the sample size, *p* is the estimated proportion, *z* is the confidence level (usually 1.96 for 95% confidence level), and *e* is the desired level of precision. The prevalence of raised blood pressure among the adult population according to the study done by Ayele Simachew and Abdurehman Kelifa, [16] in Bahir Dar city administration was 16.45%. As a result, the sample size of teachers included in this study by substituting 0.1645 for *p* was calculated as follows:

*n*=(1.96 × 1.96 × 0.1645(1 − 0.1645)/0.05 × 0.05=211) participants.

To compensate for a nonresponse rate, 5% of the calculated sample had been added and the result was (211 × 0.05) + 211 = 222. The proportional sample size taken from each secondary school is indicated in S_1.

### 2.4. Inclusion and Exclusion Criteria

Secondary school teachers in the city administration except for pregnant women and those teachers who were severely sick during the time of data collection were included in the study.

### 2.5. Study Variables

Hypertension status was the outcome (dependent) variable, and the independent (predictor) variables were sociodemographic characteristics (sex, age, marital status, religion, educational status, and income); behavioral factors (alcohol consumption, smoking, khat chewing, physical activity, feeding styles, and stress); and other factors (body mass index, waist circumference, self-reported family history of hypertension, and self-reported diabetes mellitus).

### 2.6. Blood Pressure Measurement

The prevalence of hypertension in this study was determined using the systolic and diastolic blood pressure measurements taken during the data collection time and those teachers who were under hypertension medication during the survey time. The prevalence of hypertension in this study refers to the number of respondents whose average value of SBP was above or equal to 140 mmHg and/or DBP was greater than or equal to 90 mmHg during screening time. Teachers whose average BP was less than or equal to 140/90 mmHg and under hypertension medication (taking antihypertensive drugs) during the time of data collection were considered hypertensive.

BP measurement was conducted based on the WHO guidelines for the management of hypertension [28]. Blood pressure was measured in a sitting position using a digital BP measuring device (Omron M3-Automatic Upper Arm Blood Pressure Monitor). The purpose of the measuring device was to determine the proportion of the study population with raised blood pressure. Two consecutive BP measurements were taken from the 222 respondents. BP was measured while both arms were placed on the table with the palm facing upwards. The subjects of the study remained resting for at least five minutes before the first measurement at the data collection center, and it had been made sure that they should not take caffeine and alcohol and should not do physical activity/exercise for at least 30 minutes before the BP measurement. The second BP measurement had been taken after five and more minutes' interval from the first measurement. The averages of the two readings of PB were used for analysis. The measurements were taken from 8:00 AM to 2:00 PM in schools in a private class.

### 2.7. Anthropometric Measurement

Anthropometric physical measurements such as weight, height, and waist circumference (S_2) were conducted for the 222 teachers to estimate the prevalence of overweight and obesity in the study population by excluding pregnant women. The weight and height data were used to compute BMI (S_3). The measurement values of weight and height were calculated using the BMI formula. BMI and WC were calculated to determine the obesity and overweight of the respondents. A digital personal weighing scale for weight measurement and standardized height and waist circumference measuring devices were used. Weight was measured to the nearest 0.1 kg and height to the nearest 0.1 cm in the standing position using a portable height meter. The weight measuring scale was checked and adjusted at zero levels between each measurement. The BMI of respondents was calculated as weight in kg divided by height in meters squared. Waist circumference was measured by using a nonstretching tape meter by placing a tape measure around the abdomen at the midpoint between the lower margin of the last palpable rib and the top of the iliac crest of the hipbone.

### 2.8. Questionnaire Survey

Data were collected by a structured questionnaire consisting of close-ended questions and physical measurements. The purpose of the questionnaire was to gather selected sociodemographic and behavioral characteristics of secondary school teachers from a representative sample (S_4). Anthropometric measurement spaces that were filled by a health officer were included in the questionnaire. The questionnaire was prepared in English language, and translation into Amharic was not needed since all the respondents were able to read and write the English language.

Private measurement areas or classes were prepared in the schools by school administers for BP, BMI, and WC measurements. The questionnaires were handed directly to the participants with clear instructions on how to complete the questionnaires. Finally, the filled questionnaires were collected by the investigator and other persons assigned by the investigator. The confidentiality of the findings was strictly maintained on all collected data.

### 2.9. Data Analysis

The collected data were edited, checked, and cleared manually. The edited, checked, and cleared data were coded and encoded into a computer and analyzed using SPSS Windows Version 21. Descriptive analysis like percentages, frequencies, proportions, and measures of dispersions with 95% confidence intervals for the prevalence estimates was employed to describe sociodemographic, behavioral, and other variables included in the study. The presence or absence of a significant association between independent and dependent/outcome variables was determined in the study by different statistical methods. The authors determined the association between hypertension and the sociodemographic, behavioral, dietary, and other factors by the Chi-square test of association (S_5). Then, we used the independent variables significant at 25% in the final multivariate logistic regression analysis. The presence of a statistical association between dependent and independent variables was also assessed using univariate and multivariate logistic regression analysis. *p*-values of <0.05 were considered statistically significant. Results were summarized and presented in tables.

### 2.10. Ethical Considerations

Ethical clearance was obtained from the ethical review committee of Science College of Bahir Dar University (S_6). Depending on this clearance paper, permission was obtained from the Bahir Dar city administration education department. The city administration education office wrote a permission letter for the secondary schools to get permission to conduct the study. The secondary schools' administration and teachers got an explanation about the objective of the study. Before collecting the data, informed written consent was obtained from all the study participants. Finally, the prevalence of hypertension on secondary school teachers of Bahir Dar city administration was reported to the city education and health offices to take future control and intervention measures.

### 2.11. Operational Definitions

For this study, the following definitions were given for terms and clauses.

#### 2.11.1. Physically Active

Respondents who did at least 150 minutes of moderate-intensity aerobic physical activity throughout the week or did at least 75 minutes of vigorous-intensity aerobic physical activity throughout the week or an equivalent combination of moderate- and vigorous-intensity activity were considered as physically active [29].

#### 2.11.2. Body Mass Index (BMI)

It is a person's weight in kilograms (kg) divided by his or her height in meters squared (kg/m^2^) [30]. It is classified as underweight (<18·5 kg/m^2^), normal weight (18·5–24·9 kg/m^2^), overweight (25·0–29·9 kg/m^2^), or obese (≥30·0 kg/m^2^) [31].

#### 2.11.3. Abdominal Obesity

It is the waist circumference (WC) ≥ 90 cm for men and ≥80 cm for women [32].

#### 2.11.4. Family History of Hypertension

It is defined as either parent indicating on the submitted questionnaire that they were hypertensive (diagnosed by a physician and/or on antihypertensive drugs) or had a family history of hypertension [33].

#### 2.11.5. Alcohol Use

It is defined as a self-reported intake of at least one dose in the previous 30 days; alcohol abuse was defined as four or more doses on at least one occasion during the same period [34].

#### 2.11.6. Khat Chewing

It is the practice of chewing khat leaves by the worker at least once per week [35].

#### 2.11.7. Cigarette Smoking

It is the regular inhalation of the gases and hydrocarbon vapors generated by slowly burning cigarettes [35].

#### 2.11.8. Current Smoker

A current smoker is an adult who has smoked 100 cigarettes in his or her lifetime and who currently smokes cigarettes [36].

#### 2.11.9. Nonsmoker

Someone who has not smoked greater than 100 cigarettes in a lifetime and does not currently smoke [36].

## 3. Results

### 3.1. Sociodemographic Characteristics of the Study Participants

Data were collected from 222 secondary school teachers who were randomly selected from the ten government-owned secondary schools in Bahir Dar city administration. All sample teachers voluntarily responded to the structured questionnaires and to participate in the anthropometric and blood pressure physical measurements. Due to this, the overall response rate was 100%. One hundred and forty-nine (67.1%) of the study participants were men and the rest 73 (32.9%) women with a male-to-female ratio of 2.04:1. The majority of the study participants, 139 (62.6%), were in the age category of 41 to 60 years. About 83 (37.4%) of the study participants were in the age category of 20–40 years. The majority of the teachers (52.3%) were in the middle-income category earning between 6001 to 10,000 Ethiopian Birr ($160.52–$267.49) per month.

All the respondents of the study had completed a tertiary level of education. About 142 (64%) were first-degree and the rest 80 (36%) were second-degree holders. In terms of religion, the majority of the study participants, 199 (89.6%), were Orthodox Christians, 13 (5.9%) were protestants, and the rest 10 (4.5%) were Muslims. One hundred and sixty-four (73.9%) of the study participants were married. The majority (83%) of the study participants were urban inhabitants whereas about 17% were semiurban/suburban dwellers. There were no rural residents among the subjects of the study. The detailed sociodemographic characteristics of the study participants are presented in Table 1.

### 3.2. The Prevalence of Hypertension in the Study Population

According to the data obtained from the study, the average SBP and DBP were 122.23 (±SD 18.3) mmHg and 81.75 (±SD 9.84) mmHg, respectively. The minimum and maximum values of SBP were 84.0 and 189.0 mmHg and those of DBP were 57.0 and 112.5 mmHg, respectively. Based on the systolic BP, 2 (0.9%) of the study participants were hypotensive, 107 (48.2%) normal, 72 (32.4%) prehypertensive, and 41 (18.5%) hypertensive (Table 2). According to the diastolic pressure, one individual (0.4%) was hypotensive, 97 (43.7%) normal, 75 (33.8%) prehypertensive, and 49 (22.1%) hypertensive. Among the 222 respondents, 31 (13.96%) were both systolic and diastolic hypertensive, 10 (4.5%) had isolated systolic hypertension (≥140/< 90 mmHg), and 18 (8.1%) had isolated diastolic hypertension (<140/≥ 90 mmHg). From the hypertensive teachers, 48 (21.62%) had stage I hypertension and 11(4.95%) stage II hypertension. Six of the known hypertensive study participants who were taking antihypertensive drugs (S_7) had controlled their elevated BP during the time of data collection.

### 3.3. The prevalence of Hypertension in the Study Schools

As displayed in Table 3, the prevalence of hypertension in the city administration government-owned secondary schools differs from school to school. The overall prevalence of hypertension in the study population was 65 (29.28%). It ranged from 6.7% in Zeghie secondary school to 42.9% in Diaspora secondary school. Diaspora (42.9%), Bahir Dar preparatory (37.5%), Ghion (35.7%), Fasilo (34.5%), Tana Haik (29.4%), Meshenti (27.8%), and Shum Abo (25%) had greater than the average prevalence whereas Ethio–Japanese, Shum Abo, Meshenti, and Tis Abay secondary schools had less than the average hypertension prevalence in the city.

### 3.4. Factors Associated with the Development of Hypertension in the Study Population

After adjusting for possible confounders in the multivariate logistic regression analysis, study participants who were from age categories 41 to 60 years were 2.51 times (AOR: 2.506; 95*%* CI: 1.103, 5.694; and *p*=0.028) more at risk of developing hypertension compared to those participants who were from 20 to 40 years of age. Study participants who had self-reported diabetes mellitus (DM) were 8.59 times (AOR: 8.595; 95% CI: 2.795, 26.424; and *p* < 0.001) more likely to be hypertensive compared to those who were not diabetic. Having a family history of hypertension were 3.39 times (AOR: 3.387; 95% CI: 1.579, 7.265; and *p*=0.002) at greater risk of hypertension compared to those who had not a family history of hypertension.

Khat chewers were five times more likely to be hypertensive than non-khat chewers (AOR: 5.426; 95% CI: 1.811, 16.256; and *p*=0.003). Participants who did not undertake physical activities of 150 minutes or more per week had five times more likely to develop hypertension as compared to those who participated in such amount of physical activities (AOR: 5.212; 95% CI: 1.974–13.763; and *p*=0.001). Those study participants who had repeated stress in their lifestyles were three times more likely to be hypertensive compared to those who were not stressed (AOR: 3.027; 95% CI: 1.404, 6.527; and *p*=0.005) (Table 4). Even though not statistically significant after adjusted for confounders in the adjusted odds ratio, individuals with BMI ≥25 kg/m^2^ were almost two times more likely to become hypertensive as compared to those with BMI <25 kg/m^2^ (AOR: 1.810; 95% CI: 0.829, 3.951; and *p*=0.136).

## 4. Discussion

This institutional-based cross-sectional study done on secondary school teachers in Bahir Dar city administration, northwest Ethiopia, showed an overall prevalence of hypertension of about 29.28%. The prevalence of hypertension in this study was more or less consistent with the community-based studies done in Hosaena town, Southern Ethiopia (30%) [37]; Gondar town, northwest Ethiopia (28.3%) [38]; Addis Ababa city (30.2%) [39]; USA (29.3%) [40]; Zambia (31.8%) [41]; African prevalence (30%) [42]; Addis Ababa (27.3%) [43]; Arba Minch town, southern Ethiopia (27.8%) [44]; and Kenya (30%) [45].

The prevalence of hypertension in this study was lower than the national prevalence (35.2%) [46], the African subregional prevalence (47.5%) [47], and the prevalence of the studies done in Nigeria (36.6%) [48], in Bangladesh (52%) [49], in Zambia (40%) [50], in Ghana (42.4%), and in South Africa (46%) [51]. However, it was higher than that of the studies done in Northern Ethiopia (18.1%) [52], in Bahir Dar, northwest Ethiopia (16.45%) [16], in Jimma, Southwest Ethiopia (13%) [53], in Addis Ababa (21%) [17], in Durame town, Southern Ethiopia (22.4%) [54], and in Owerri, Nigeria (12.4%) [55].

Variations in the prevalence of this study and other studies might be attributed to the differences in sociodemographic characteristics, sample size differences, lifestyle, feeding habits, differences of study settings, and other variations of the study subjects. For example, in the Zambian study mentioned above, 22% of the study subjects were cigarette smokers and 63% were alcohol consumers, which is very high compared to the subjects of the present study (8.56% smokers and 52.25% alcohol consumers). Furthermore, the subjects of this study were secondary school teachers who were educated. However, subjects of other studies were from the general adult population. In addition, other studies may be conducted both in urban and rural settings compared to the present study which was conducted among urban dwellers.

The prevalence of hypertension in the present study was higher among male participants than female participants. The higher occurrence of hypertension among men may be attributed to the large number of men engaged in risky behaviors such as alcohol consumption, smoking, and khat chewing than women. The level of hypertension is raised with the rise of age. Teachers aged 41 to 60 years were two and half times more hypertensive than teachers aged 20 to 40 years. This finding is in line with the community-based studies conducted in Gondar [38], in Bedele [56], and in Durame town [54] in Ethiopia. Aging leads to less physical activity, increases peripheral resistance, and results in hormonal changes. These situations in turn cause high blood pressure [57]. Older persons have a four times higher risk of hypertension than younger persons. In 2008, hypertension in adults older than 25 years of age was 40% and accounted for 12.8% of the global deaths [18].

Self-reported diabetes mellitus was also another factor for the higher prevalence of hypertension in the present study. Teachers who self-reported as diabetic patients were 8.595 times (AOR: 8.595; 95% CI: 2.795–26.424) more likely to be hypertensive than teachers who did not. This result is in agreement with the study findings in northwest Ethiopia (AOR: 4.15; 95% CI: 1.77–9.72) [38] and Addis Ababa (AOR: 13.56; 95% CI: 6.91–26.96) [43]. The high risk of hypertension among diabetic individuals could be due to increased peripheral vascular resistance and the fact that both diseases share common risk factors. Both diabetes and hypertension may cause each other. People with DM are more likely to develop hypertension due to the hardening of the arteries [58].

In this study, individuals with a positive family history of hypertension were more likely to be hypertensive. Those who had a family history of hypertension were 3.387 times (AOR: 3.387; 95% CI: 1.579–7.281; and *p*=0.002) more likely to be hypertensive than those who did not have. The finding was in line with the studies conducted in Gondar, northwest Ethiopia (AOR: 2.71; 95% CI: 1.37–5.36) [38], and in Debre Markos town (AOR: 4.38; 95% CI: 1.985–12.015; and *p* < 0.01) [59]. This may be because family members may share similar lifestyles and genetic factors since participants with a family history of hypertension have the same genetic component and families tend to share the same lifestyle choice and behavior [60].

We also reported a positive association between khat chewing and hypertension. Khat chewers were 5.426 times (AOR: 5.426; 95% CI: 1.811–16.256; and *p*=0.003) at risk of hypertension than non-khat chewers. This finding was similar with the findings of the studies done in Addis Ababa (AOR: 5.0; 95% CI: 1.91–13.06; and *p*=0.001) [61] and in Jimma town, southwest Ethiopia (AOR: 1.50; 95% CI: 1.45–2.87; and *p* < 0.001) [62]. The leaves of the khat plant contain amphetamine-like compounds (cathinone and cathine) which increase vasoconstriction and heart rate which in turn increase blood pressure [63]. According to a study done by Geta et al. [64], the mean systolic and diastolic blood pressures were significantly higher among khat chewers compared with non-khat chewers (122.22 ± 17.84 mmHg vs. 109.25 ± 15.08 mmHg) and (75.71 ± 12.21 mmHg vs. 68.08 ± 11.31 mmHg), respectively.

Physical inactivity was identified as another factor associated with hypertension in this study. Physically inactive teachers were 5.212 times (AOR: 5.212; 95% CI: 1.974–13.763; and *p*=0.001) more likely to be hypertensive than physically active teachers. This result was similar to that of the studies done in Bedele town, southwest Ethiopia (AOR: 3.10; 95% CI: 0.97–9.97) [56], and Gondar, northwest Ethiopia (AOR: 2.86; 95% CI: 1.5–7.12) [38]. Regular exercise reduces the risk of many diseases including hypertension [65]. In a study conducted in India, sedentary people were 20 to 50% at a high risk of hypertension than physically active people [66]. A report from northwest Ethiopia also showed that people who did not walk for at least 10 minutes daily were about three times more likely to be hypertensive than their counterparts [38].

The stress level of the study participants was another most important factor of hypertension in this study. Secondary school teachers with stressful conditions were 3.027 times at higher risk of hypertension than those who were not repeatedly stressed (AOR: 3.027; 95% CI: 1.404–6.527; and *p*=0.005). This result was in line with a similar study done in Arba Minch town, Southern Ethiopia (AOR: 12.3; 95% CI: 4.9–30.0; and *p* < 0.001) [44]. Stress increases the heart rate and can raise blood pressure over a while. Consequently, it precipitates in heart attack and stroke [67]. Stress can cause hypertension through frequent blood pressure elevations as well as by stimulation of the nervous system to produce large amounts of vasoconstriction hormones that increase blood pressure. Overall, studies show that stress does not directly cause hypertension, but can have an effect on its development [68]. From the researcher's own experience, the teaching profession is a highly stressful occupation. The major sources of stress for secondary school teachers may be the curriculum, pupils, school authority, society, teaching environment, less income, and high living costs.

Higher BMI was evidenced as another risk factor for hypertension. Those secondary school teachers who were categorized as overweight and obese (≥25 kg/m^2^) were almost two times (AOR: 2.161; 95% CI: 0.916–5.099; and *p*=0.078) more likely to be hypertensive compared to those whose BMIs were <25 kg/m^2^ even if the association was not statistically significant in the multivariate logistic regression analysis. This result is consistent with studies conducted in Durame town, Southern Ethiopia (AOR: 15.7; 95% CI: 7.89–31.21) [54] and Debre Markos town in northwest Ethiopia (AOR: 4.70; 95% CI: 1.99–11.065; and *p* < 0.001) [59]. The observed relationship between overweight, obesity, and hypertension could be due to the differences in dietary habits and physical inactivity of the study participants. As BMI level increases the risk of chronic diseases increases and an increased level of BMI indicates the accumulation of fat tissue in the human body, this might be because excess weight increases blood cholesterol and triglyceride levels and lowers high-density lipoprotein levels [59].

## 5. Conclusion

The overall prevalence of hypertension among the study participants in this study was 29.28%. Seventy-eight (35.1%) of the study participants were in the prehypertension stage, which increases the overall future risk of hypertension. Among the hypertensive teachers, 39 (60%) did not know their hypertension status before this study. Seventeen (26.15%) of the hypertensive teachers were under hypertension medication (taking antihypertensive drugs), and six of these have controlled their elevated blood pressure. Aging, self-reported diabetes mellitus, family history of hypertension, khat chewing, physical inactivity, and stressful conditions were the risk factor associated with hypertension among the secondary school teachers of Bahir Dar city administration. Modification of lifestyles like controlling diabetes mellitus, healthy feeding, avoiding khat chewing, doing physical exercise, avoiding stressful conditions, controlling body mass index, and making regular checkups are highly recommended to control and prevent hypertension.

## 6. Significance of the Study

The findings of this study will add to the literature about the prevalence of hypertension and associated risk factors among secondary school teachers. Data on the prevalence of hypertension can guide policymakers to design proactive and workable programs for managing and controlling the condition. The findings of the study will also contribute to academic knowledge and add to ongoing research efforts on hypertension. Valuable information about the prevalence of hypertension and associated risk factors will be generated in the study area. It will also serve as a source of relevant information for those who want to carry out further investigation on hypertension. Baseline information about hypertension will be provided to the local health centers or other concerned agencies to develop appropriate prevention methods to control hypertension in the study area.

## 7. Limitations of the Study

The findings from this study may not apply to the general population of the city administration because the secondary school teachers are not representatives of the various aspects of the population including age, sex distribution, occupation, educational level, social status, and location of residence. The study may not be free from recall and social desirability biases from self-reported variables like self-reported DM and family history of hypertension.

## Figures and Tables

**Table 1:** Sociodemographic characteristics of the study participants, Bahir Dar, northwest Ethiopia, January 2020 (*n* = 222).

Characteristics	Category	Frequency	Percent
Sex	Male	149	67.1
	Female	73	32.9

Age	20–40	83	37.4
	41–60	139	62.6

Monthly income	≤3000 Ethiopian Birr (≤$80.25)	5	2.3
	3001–6000 Ethiopian Birr ($80.27–$160.49)	91	41
	6001–10000 Ethiopian Birr ($160.52–$267.49)	116	52.3
	Above 10000 Ethiopian Birr (>267.49)	10	4.5

Level of education	Bachelor's degree	142	64
	Master's degree	80	36

Religion	Orthodox	199	89.6
	Muslim	10	4.5
	Protestant	13	5.9

Marital status	Single	38	17.1
	Married	164	73.9
	Divorced	16	7.2
	Widowed	4	1.8

Residence	Urban	185	83.3
	Suburban	37	16.7

**Table 2:** Distribution and classification of blood pressure in the study population, Bahir Dar, northwest Ethiopia, January 2020 (*n* = 222).

Variables	Category	Frequency	Percent
SBP	<90 mmHg (hypotensive)	2	0.9
	90–119.99 mmHg (normal)	107	48.2
	120–139.99 (prehypertensive)	72	32.4
	≥140 mmHg (hypertensive)	41	18.5
	*Mean systolic*	**122.23**	

DBP	<60 mmHg (hypotensive)	1	0.4
	60–79 mmHg (normal)	97	43.7
	80–89.99 (prehypertensive)	75	33.8
	≥90 mmHg (hypertensive)	49	22.1
	*Mean diastolic*	**81.75**	

Magnitude of hypertension	Controlled hypertension (by taking antihypertensive drugs)	6	2.7
	Hypertensive by physical measurement	59	26.58
	Total	65	29.28

Isolated BP-values	Isolated systolic hypertension	10	4.5
	Isolated diastolic hypertension	18	8.1
	Total	28	12.6

Stages of hypertension	Stage I	48	21.62
	Stage II	11	4.95
	Total	59	26.57

**Table 3:** Prevalence of hypertension in the study schools.

School name	Hypertensive			Nonhypertensive			Hypertension prevalence in %
	Male	Female	Total	Male	Female	Total	
Ghion ss^*∗*^	11	4	15	13	14	27	35.7
Tana Haik ss	7	3	10	17	7	24	29.4
Fasilo ss	8	2	10	10	9	19	34.5
Bahir Dar preps^*∗*^	10	2	12	15	5	20	37.5
Ethio-Japanese ss	2	0	2	9	3	12	14.3
Shum abo ss	2	1	3	6	3	9	25
Diaspora ss	3	3	6	6	2	8	42.9
Zegie ss	0	1	1	11	3	14	6.7
Meshenti ss	3	2	5	8	5	13	27.8
Tis Abay ss	1	0	1	7	4	11	8.33
Total	47	18	65	102	55	157	29.28

*Note.* ss = secondary school; preps = preparatory school.

**Table 4:** Multivariate logistic regression analysis for the risk factors associated with hypertension, among Bahir Dar secondary school teachers, northwest Ethiopia, January 2020 (*n* = 222).

Variables	Category	Hypertensive	Nonhypertensive	Crude OR (95% CI)	*p* value	Adjusted OR (95% CI)	*p* value
		*n* (%)	*n* (%)				
Age	20–40	15 (6.76)	68 (30.63)	1	0.005^*∗*^	1	0.028^*∗*^
	41–50	50 (22.52)	89 (40.09)	2.547 (1.319–4.916)		2.506 (1.103–5.694)	

Diabetes mellitus	Yes	16 (7.21)	6 (2.7)	8.218 (3.047–22.161)	<0.001^*∗*^	8.595 (2.795–26.424)	<0.001^*∗*^
	No	49 (22.07)	151 (68.02)	1		1	

Family history of hypertension	Yes	33 (14.86)	35 (15.77)	3.595 (1.944–6.646)	<0.001^*∗*^	3.387 (1.579–7.285)	0.002^*∗*^
	No	32 (14.41)	122 (54.95)	1		1	

Khat chewing	Yes	14 (6.3)	11 (4.95)	3.643 (1.555–8.538)	0.003^*∗*^	5.426 (1.811–16.256)	0.003^*∗*^
	No	51 (22.97)	146 (65.77)	1		1	

Physical activity	Inactive	55 (24.77)	98 (44.14)	3.311 (1.569–6.990)	0.002^*∗*^	5.212 (1.974–13.763)	0.001^*∗*^
	Active	10 (4.5)	59 (26.58)	1		1	

Repeated stress	Yes	29 (13.06)	33 (14.86)	3.027 (1.625–5.637)	<0.001^*∗*^	3.027 (1.404–6.527)	0.005^*∗*^
	No	36 (16.22)	124 (55.86)	1		1	

BMI	<25 kg/m2	34 (15.32)	118 (53.15)	1	0.001^*∗*^	1	0.136
	≥25 kg/m2	31 (13.96)	39 (17.57)	2.759 (1.504–5.060)		1.810 (0.829–3.951)	

^*∗*^Statistically significant at a *p* value of <0.05.

## Data Availability

All the data sets generated and analyzed during the study are included the text.

## Abbreviations

AOR: Adjusted odds ratio
BMI: Body mass index
BP: Blood pressure
CDC: Center for Disease Control and Prevention
CI: Confidence interval
COR: Crude odds ratio
CVDs: Cardiovascular diseases
DBP: Diastolic blood pressure
DM: Diabetes mellitus
mmHg: millimeters of mercury
NCDs: Noncommunicable diseases
SBP: Systolic blood pressure
SPSS: Statistical Package for the Social Sciences
ss: Secondary school
WC: Waist circumference
WHO: World Health Organization.

## Supplementary Materials

Supplementary Materials
*S_1*: total sample size allocation by secondary school. *S_2*: waist circumference status of the study subjects by gender. *S_3*: body mass index status of the study subjects by gender. *S_4*: behavioral, dietary, and other characteristics of the study subjects, Bahir Dar, northwest Ethiopia, January 2020 (*n* = 222). *S_5*: chi-square association between sociodemographic, behavioral, dietary, and other factors and hypertension among Bahir Dar secondary school teachers, northwest Ethiopia, January 2020 (*n* = 222). *S_6*: ethical clearance. *S_7*: awareness, diagnosis, treatment, and checkup practice of hypertension in the study population, Bahir Dar, northwest Ethiopia, January 2020 (*n* = 222).
