# Supplementary material for: The Prevalence of Hypertension and Associated Risk Factors among Secondary School Teachers in Bahir Dar City Administration, Northwest Ethiopia
Source: Int J Hypertens. 2021 Apr 16;2021:5525802. doi: 10.1155/2021/5525802 (PMC8064782; doi:10.1155/2021/5525802)
Supplement: Supplementary Materials — S_1: total sample size allocation by secondary school. S_2: waist circumference status of the study subjects by gender. S_3: body mass index status of the study subjects by gender. S_4: behavioral, dietary, and other characteristics of the study subjects, Bahir Dar, northwest Ethiopia, January 2020 (n = 222). S_5: chi-square association between sociodemographic, behavioral, dietary, and other factors and hypertension among Bahir Dar secondary school teachers, northwest Ethiopia, January 2020 (n = 222). S_6: ethical clearance. S_7: awareness, diagnosis, treatment, and checkup practice of hypertension in the study population, Bahir Dar, northwest Ethiopia, January 2020 (n = 222). [file 5525802.f1.docx]

S_1. Total sample size allocation by secondary school

| **S.N** | **Name of Secondary schools** | **Number of all Teachers** | | | **Randomly Selected Sample Size** | | |
| --- | --- | --- | --- | --- | --- | --- | --- |
|  |  | **Male** | **Female** | **Total** | **Male** | **Female** | **Total** |
| 1 | Ghion | 88 | 47 | 135 | 24 | 18 | 42 |
| 2 | Tana Haik | 79 | 35 | 114 | 24 | 10 | 34 |
| 3 | Fasilo | 62 | 32 | 94 | 18 | 11 | 29 |
| 4 | Bahir Dar Preparatory | 79 | 20 | 99 | 25 | 7 | 32 |
| 5 | Diaspora | 27 | 18 | 45 | 9 | 5 | 14 |
| 6 | Ethio-Japan | 34 | 15 | 49 | 11 | 3 | 14 |
| 7 | Shum Abo | 29 | 7 | 36 | 8 | 4 | 12 |
| 8 | Zegie | 31 | 8 | 39 | 11 | 4 | 15 |
| 9 | Meshenti | 37 | 16 | 53 | 11 | 7 | 18 |
| 10 | Tis Abay | 26 | 20 | 46 | 8 | 4 | 12 |
| Total | | 492 | 218 | 710 | 149 | 73 | 222 |

S_2. Waist circumference Status of the Study Subjects by gender

| Gender | Waist Circumference | | Total  No. (*%*) |
| --- | --- | --- | --- |
|  | Male < 94cm/ women < 80cm /Normal/  No. (*%*) | Male ≥94 cm /women ≥ 80 cm /Obese and Very obese/  No. (*%*) |  |
| Male | 115 (77.2) | 34 (22.8) | 149 (100) |
| Female | 24 (32.9) | 49 (67.1) | 73 (100) |
| Total | 139 (62.6) | 83 (37.4) | 222 (100) |

S_3. Body Mass Index Status of the Study Subjects by gender

| Gender | Body mass index status (kg/m^2^) | | | | Total  No. (*%*) |
| --- | --- | --- | --- | --- | --- |
|  | < 18.5 (underweight)  No. (*%*) | 18 .5 - 24.99 (Normal)  No. (*%*) | 25 - 29.99 (Over weight)  No. (*%*) | ≥ 30 (Obese)  No. (*%*) |  |
| Male | 5 (3.4) | 103 (69.1) | 38 (25.5) | 3 (2.0) | 149 (100) |
| Female | 6 (8.2) | 38 (52.1) | 24 (32.9) | 5 (6.8) | 73 (100) |
| Total | 11 (5) | 141 (63.5) | 62 (27.9) | 8 (3.6) | 222 (100) |

|  |
| --- |
|  |

S_4. Behavioral, Dietary, and Other Characteristics of the study subjects, Bahir Dar, Northwest Ethiopia, January 2020, (n= 222)

| **Characteristics (Variables)** | **Category** | **Frequency** | **Percent** |
| --- | --- | --- | --- |
| Alcohol drinking | Yes | 113 | 50.90 |
|  | No | 109 | 49.1 |
| Cigarette Smoking | Smoker | 19 | 8.56 |
|  | Non-smoker | 203 | 91.44 |
| Khat chewing | Yes | 25 | 11.26 |
|  | No | 197 | 88.74 |
| Physical activity | Inactive | 153 | 68.9 |
|  | Active | 69 | 31.1 |
| Habit of eating vegetables and fruits | Yes | 162 | 73 |
|  | No | 60 | 27 |
| Habit of eating fatty and oily food | Yes | 129 | 58.1 |
|  | No | 93 | 41.9 |
| The habit of eating extra salt | Yes | 80 | 36 |
|  | No | 142 | 64 |
| Repeated stress | Yes | 62 | 27.9 |
|  | No | 160 | 72.1 |
| Diabetes mellitus | Yes  No | 22  200 | 9.91  90.09 |
| History of hypertension  in the family | Yes  No | 68  154 | 30.63  69.37 |

S_5. Chi-square association between socio-demographic, behavioral, dietary and other factors and hypertension among Bahir Dar secondary school teachers, Northwestern Ethiopia, January 2020, (n= 222)

| **Variables** | **Category** | **Hypertensive**  **N (*%*)** | **Non hypertensive**  **N (*%*)** | **Total (*%*)** | **X^2^** | **P-value** |
| --- | --- | --- | --- | --- | --- | --- |
| Gender | Male | 47 (21.17) | 102 (45.95) | 149 (67.12) | 1.122 | 0.289 |
|  | Female | 18 (8.11) | 55 (24.77) | 73 (32.88) |  |  |
| Age | 20 – 40 | 15 ( 6.76) | 68 ( 30.63) | 83 (37.39) | 8.041 | 0.005* |
|  | 41 – 60 | 50 (22.52) | 89 (40.09) | 139 (62.61) |  |  |
| Income | Below 3000 birr | 2 (0.9) | 3 (1.35) | 5 (2.25) | 7.104 | 0.069 |
|  | 3001 - 6000 birr | 20 (9) | 71 (31.98) | 91 (40.98) |  |  |
|  | 6001 - 10000 birr | 43 (19.37) | 83 (37.39) | 126 (56.76) |  |  |
| Level of  Education | Bachelor Degree | 38 (17.12) | 104 (46.85) | 142 (63.97) | 1.207 | 0.272 |
|  | Master’s Degree | 27 (12.16) | 53 (23.87) | 80 (36.04) |  |  |
| Religion | Orthodox | 54 (24.32) | 145 (65.32) | 199 (89.64) | 5.529 | 0.63 |
|  | Muslim | 6 (2.7) | 4 (1.8) | 10 (4.5) |  |  |
|  | Protestant | 5 (2.25) | 8 (3.6) | 13 (5.85) |  |  |
| Marital status | Single | 9 (4.05) | 29 (13.06) | 38 (17.12) | 4.841 | 0.184 |
|  | Married | 46 (20.72) | 118 (53.15) | 164 (73.87) |  |  |
|  | Divorced | 8 (3.6) | 8 (3.6) | 16 (7.2) |  |  |
|  | Widowed | 2 (0.9) | 2 (0.9) | 4 (1.8) |  |  |
| Residence | Urban | 57 (25.68) | 128 (57.66) | 185 (83.34) | 1.257 | 0.262 |
|  | Suburban | 8 (3.6) | 29 (13.06) | 37 (16.67) |  |  |
| Diabetes mellitus | Yes | 16 (7.21) | 6 (2.7) | 22 (9.91) | 22.263 | < 0.001* |
|  | No | 49 (22.07) | 151 (68.02) | 200 (90.09) |  |  |
| Family history  of hypertension | Yes | 33 (14.86) | 35 (15.77) | 68 (30.63) | 17.543 | < 0.001* |
|  | No | 32 (14.41) | 122 (54.95) | 154 (69.36) |  |  |
| Drinking Alcohol | Yes | 38 (17.12) | 75 (33.78) | 113 (50.9) | 2.102 | 0.147 |
|  | No | 27 (12.16) | 82 (36.94) | 109 (49.1) |  |  |
| Cigarette Smoking | Smoker | 8 (3.6) | 11 (4.95) | 19 (8.55) | 1.651 | 0.199 |
|  | Non-smoker | 57 (25.68) | 146 (65.77) | 203 (91.45) |  |  |
| Khat chewing | Yes | 14 (6.3) | 11 (4.95) | 25 (11.26) | 9.714 | 0.002* |
|  | No | 51 (22.97) | 146 (65.77) | 197 (88.74) |  |  |
| Physical activity | Inactive | 55 (24.77) | 98 (44.14) | 153 (68.92) | 10.571 | 0.001* |
|  | Active | 10 (4.5) | 59 (26.58) | 69 (31.08) |  |  |
| Eating veg. and fruits | No | 21 (9.46) | 39 (17.57) | 60 (27.03) | 1.300 | 0.254 |
|  | Yes | 44 (19.82) | 118 (53.15) | 162 (72.97) |  |  |
| Eating fatty and oily food | Yes | 39 (17.57) | 90 (40.54) | 129 (58.11) | 0.135 | 0.713 |
|  | No | 26 (11.71) | 67 (30.18) | 93 (41.89) |  |  |
| Eating extra salt | Yes | 24 (10.81) | 56 (25.23) | 80 (36.04) | 0.031 | 0.859 |
|  | No | 41 (18.47) | 101 (45.5) | 142 (63.97) |  |  |
| Repeated stress | Yes | 29 (13.06) | 33 (14.86) | 62 (27.92) | 12.716 | < 0.001* |
|  | No | 36 (16.22) | 124 (55.86) | 160 (72.08) |  |  |
| BMI | < 25 kg/m2 | 34 (15.32) | 118 (53.15) | 152 (68.47) | 11.119 | 0.001* |
|  | ≥ 25kg/m2 | 31 (13.96) | 39(17.57) | 70(31.53) |  |  |
| WC | ≥ 94 cm /≥ 80 cm | 26 (11.71) | 57(25.67) | 83(37.38) | 0.268 | 0.605 |
|  | < 94 cm/< 80 cm | 39(17.56) | 100 (45.05) | 139 (62.61) |  |  |

**Statistically significant at a p-value of < 0.05*

# S_6. Ethical clearance

S_7. Awareness, diagnosis, treatment, and checkup practice of hypertension in the study population, Bahir Dar, Northwestern Ethiopia, January 2020. (n= 222)

| **Variables** | **Category** | **Frequency** | **Percent** |
| --- | --- | --- | --- |
| Do you know the definition of hypertension? | Yes  No | 179  43 | 80.6  19.4 |
| Is hypertension preventable/ controllable/ manageable? | Yes | 191 | 86 |
|  | No | 31 | 14 |
| Have you ever been checked and measured your blood pressure? | Yes | 170 | 76.6 |
|  | No | 52 | 23.4 |
| Have you ever been diagnosed in health institutions with hypertension? | Yes | 116 | 52.3 |
|  | No | 106 | 47.7 |
| Are you currently hypertensive? | Yes | 26 | 11.7 |
|  | No | 196 | 88.3 |
| Have you started anti-hypertension drugs? | Yes | 17 | 7.66 |
|  | No | 9 | 4.05 |
